# The Relationship Between Family Dynamics and Help-Seeking and Disclosure of Adolescent Self-Harm and Suicidality: A Population-Representative Study: Relation entre dynamique familiale et recherche d’aide, et dévoilement des actes d’automutilation et de la suicidalité chez les adolescents : étude représentative de la population

**DOI:** 10.1177/07067437251315526

**Published:** 2025-02-03

**Authors:** Nicole G. Hammond, Christopher Gravel, Mark A. Ferro, Hannah Landry, Marie-Claude Geoffroy, Nicole Racine, Ian Colman

**Affiliations:** 1 School of Epidemiology and Public Health, 151181 University of Ottawa, Ottawa, Canada; 2 Department of Epidemiology, Biostatistics and Occupational Health, 5620 McGill University, Montreal, Canada; 3 Department of Mathematics and Statistics, 151181 University of Ottawa, Ottawa, Canada; 4 Data Literacy Research Institute, 151181 University of Ottawa, Ottawa, Canada; 5 School of Public Health Sciences, 8430 University of Waterloo, Waterloo, Canada; 6 Department of Educational and Counselling Psychology, 5620 McGill University, Montreal, Canada; 7 Department of Psychiatry, 5620 McGill University & Douglas Mental Health University Institute, Montreal, Canada; 8 School of Psychology, 151181 University of Ottawa, Ottawa, Canada; 9 Children's Hospital of Eastern Ontario Research Institute, Ottawa, Canada; 10 Centre for Fertility and Health, Norwegian Institute of Public Health, Oslo, Norway

**Keywords:** family dynamics, parenting practices, family functioning, help-seeking, disclosure, adolescents, self-harm, suicidal ideation

## Abstract

**Background:**

Few studies have explored the potential for family dynamics to hinder or promote help-seeking and disclosure behaviours among adolescents who self-harm or experience suicidality. We sought to examine whether family dynamics may influence self-harm-related disclosure to parents or other family members and online help-seeking.

**Methods:**

We identified youths, 14–17 years, in the 2014 Ontario Child Health Study (OCHS) who self-reported past-year suicidal ideation (with or without a suicide plan or past suicide attempt[s]) and/or non-suicidal self-harm. The OCHS is a provincially representative, cross-sectional survey. The person most knowledgeable about the adolescent, usually the mother, reported family dynamics: family dysfunction and positive and negative parenting practices. We used logistic regression to generate adjusted odds ratios.

**Results:**

A total of 359 adolescents positively endorsed past-year suicidal ideation and/or non-suicidal self-harm. Disclosure and help-seeking were common (≥67.3% and ≥25.6%, respectively). Adolescents experiencing suicidal ideation and greater family dysfunction were more likely to share their suicidal thoughts with non-family compared to not telling anyone (OR = 1.09, 95% CI: 1.01 to 1.18) and were less likely to tell their parents or other family members about their suicidal thoughts when compared to non-family such as teachers, partners, or friends (OR = 0.82, 95% CI: 0.71 to 0.94). Positive parenting was not associated with any form of disclosure or online help-seeking for non-suicidal self-harm or suicidal ideation. As adolescent exposure to negative parenting increased, so did the likelihood that they would seek help online for their suicidal thoughts (OR = 1.22, 95% CI: 1.08 to 1.37). Sensitivity analyses replicated or were very similar to findings from the main models.

**Conclusion:**

We found that negative family dynamics were related to reduced sharing of suicidal thoughts with parents or other family members and greater online help-seeking. Our findings suggest that the importance of negative family dynamics to disclosure and support-seeking for adolescent suicidality may be under-recognized.

## Résumé

**Contexte:**

peu d’études ont exploré le rôle que peut jouer la dynamique familiale dans la recherche d’aide et le dévoilement des comportements chez les adolescents qui s’automutilent ou qui sont suicidaires. Nous avons cherché à déterminer si la dynamique familiale pouvait influencer le dévoilement des actes d’automutilation aux parents ou à d’autres membres de la famille, ainsi que la recherche d’aide en ligne.

**Méthodologie:**

nous avons identifié les jeunes âgés de 14 à 17 ans qui ont participé à l’Étude sur la santé des jeunes Ontariens (ESJO) de 2014 et ont déclaré avoir eu des idées suicidaires au cours de l’année écoulée (avec ou sans plan de suicide ou tentative(s) de suicide antérieure(s)) et/ou s’être livrés à des actes d’automutilation non suicidaire. L’ESJO est une enquête transversale représentative à l’échelle provinciale. La personne qui connaît le mieux l’adolescent, généralement la mère, a fait état de la dynamique familiale : famille dysfonctionnelle et pratiques parentales positives et négatives. Nous avons utilisé la régression logistique pour générer des rapports de cotes ajustés.

**Résultats:**

au total, 359 adolescents ont déclaré avoir eu des idées suicidaires et/ou s’être automutilés sans intention de se suicider au cours de l’année écoulée. Le dévoilement et la recherche d’aide étaient fréquents (≥ 67% et ≥ 25%, respectivement). Les adolescents ayant des idées suicidaires et vivant dans une famille très dysfonctionnelle étaient plus susceptibles de partager leurs idées suicidaires avec des personnes qui ne sont pas membres de leur famille que de n’en parler à personne (RC = 1.09, IC 95%: 1.01, 1.18) et étaient moins susceptibles de parler de leurs idées suicidaires à leurs parents ou à d’autres membres de leur famille qu’à des personnes qui ne sont pas membres de leur famille, telles que des enseignants, un amoureux ou une amoureuse ou des amis (RC = 0.82, IC 95%: 0.71, 0.94). La parentalité positive n’était associée à aucune forme de dévoilement ou de recherche d’aide en ligne en ce qui concerne les actes d’automutilation non suicidaire ou les idées suicidaires. Plus les adolescents sont exposés à des pratiques parentales négatives, plus ils sont susceptibles de chercher de l’aide en ligne à cause d’idées suicidaires (RC = 1.22, IC 95%: 1.08, 1.37). Les analyses de sensibilité reproduisaient les résultats des modèles principaux ou étaient très similaires à ceux-ci.

**Conclusion:**

nous avons constaté qu’une dynamique familiale négative était liée à un plus faible partage des idées suicidaires avec les parents ou d’autres membres de la famille et à une plus grande recherche d’aide en ligne. Nos résultats laissent penser que le rôle des dynamiques familiales négatives dans le dévoilement et la recherche de soutien en cas de suicidalité chez les adolescents n’est peut-être pas suffisamment reconnu.

Adolescence is a developmental period comprised of important biological and emotional changes.^1^ It is also a period of elevated risk for mental health concerns.^2^ Globally, engagement in non-suicidal self-harm, defined as deliberate bodily harm (e.g., skin cutting) but without the intent to die,^3^ is the most prevalent form of child (21.9%)^4^ and adolescent (19.5%)^5^ self-harm behaviour. Suicidal ideation is also common in childhood (7.5%)^4^ and adolescence (14.2%).^5^ As a collection of risk factors, self-injurious thoughts and behaviours increase the risk of future suicide attempts^6^ and mental disorders.^7^ Further, adolescent intentional self-harm presentation at emergency departments is rising.^8^ Over an approximately 8-year period (2009 to 2017), hospital presentations for intentional self-harm in Ontario, Canada, increased by 135%, putting substantial demand on finite resources.^8^ Increasing use of emergency services for acute self-harm has also been observed in Australia^9^ and the United States.^10^ Persons who self-harm are frequent users of emergency department services,^11^ and adolescents who self-harm are more likely to have shorter duration between recurrent self-harm-related hospital visits, die by suicide, and have greater associated healthcare costs.^12^

Approximately 33–50% of adolescents who engage in self-harm do not seek support^13^ until they require urgent care. Among those who reach out to others, informal supports such as family and friends are more frequently relied on than more formal resources (e.g., mental health professionals).^13^ The potential use of the internet as another recovery resource is complicated, but it can be used in a positive context for self-harm-related disclosure and help-seeking.^13,14^ In a recent review, positive relationships with pertinent adults in one's life (e.g., parents and teachers) were identified as help-seeking facilitators for general adolescent mental health concerns.^15^ However, few studies focused on relationship quality as a facilitator, and none addressed family functioning,^15^ such as the family's everyday ability to work cohesively and communicate.^16^ An Australian study^17^ explored positive parenting practices and observed that parental authoritativeness and social support were associated with initial intentions to seek professional mental health support but not later help-seeking behaviour.

There is a need to identify factors that may promote help-seeking among young persons who engage in self-harm.^13^ Some interventional research suggests that targeting the family unit is a means to reduce self-harm.^18,19^ However, it is unknown whether everyday family dynamics, such as family functioning and parenting practices, may promote help-seeking and disclosure. Identifying familial factors that may impede or encourage seeking support could be used to inform first-line, preventative public mental health aid. Using a provincially representative sample of adolescents in Ontario experiencing self-harming thoughts and behaviours, our study objectives were to examine relationships between family functioning and parenting practices (positive and negative) with self-harm-related disclosure and online help-seeking. We hypothesized that greater exposure to family dysfunction and negative parenting practices would be associated with a reduced likelihood of self-harm-related disclosure and online help-seeking. In comparison, greater exposure to positive parenting practices would be associated with an increased likelihood of self-harm-related disclosure and online help-seeking.

## Methods

### Data Source, Setting, and Study Population

The 2014 Ontario Child Health Study (OCHS) is a cross-sectional, provincially representative epidemiological study of Ontarians between 4 and 17 years (*n* = 10,802).^20^ The sampling frame was the 2014 Canada Child Tax Benefit, with the sample selected using complex survey methods (stratification and clustering) and simple random sampling.^20^ Data were collected in person from October 2014 to September 2015 by trained Statistics Canada interviewers, and the overall response rate was 50.8%.^20^ The person most knowledgeable about the surveyed child(ren) was most often the mother (88.3%).^20^

Youths aged 14–17 (*n* = 2,910) self-reported on sensitive items such as past-year non-suicidal self-harm (deliberately harming oneself without intending to kill oneself) and suicidal ideation (seriously considering killing oneself) (Supplemental Table S1). The sample for this study included those who positively endorsed past-year non-suicidal self-harm and/or suicidal ideation (*n* = 359).

### Measures

#### Exposures: Family Dynamics

The person most knowledgeable about the child reported on family dynamics: family dysfunction (primary exposure) and parenting practices (secondary exposure). Family dysfunction was measured using the 12-item general functioning subscale of the McMaster Family Assessment Device.^16^ The subscale provides an overall index of everyday family functioning (e.g., “making decisions is a problem for our family”^21^) and was initially validated in the 1983 OCHS.^16^ Higher scores reflect greater family dysfunction. Parenting practices were assessed using items selected or adapted from the Parent Behavior Inventory^22^ and the Parenting Scales of the National Longitudinal Survey of Children and Youth.^23,24^ Both positive (e.g., “I listen to [child's] ideas and opinions”) and negative (e.g., “whether I enforce or do not enforce a rule depends on my mood”) parenting practices over the past six months were assessed using five items, with higher scores more reflective of the respective parenting style. Cronbach's alpha (α) for the family dynamics measures suggests adequate internal consistency (family functioning α = 0.87; positive parenting α = 0.84; negative parenting α = 0.77).^24^ The correlations (*r*) among the family dynamics measures were in the expected directions (family dysfunction-negative parenting: 0.25, family dysfunction-positive parenting: −0.32, and negative-positive parenting practices: −0.09).

#### Outcomes: Disclosure and Help-Seeking Behaviour

Participants who positively endorsed non-suicidal self-harm or suicidal ideation were asked to report whether they disclosed to anyone, potentially up to 9 different persons (e.g., “parent or other family member,” “friend or partner”), that they had harmed themselves or were experiencing suicidal ideation (Supplemental Table S1). We classified respondents as having disclosed their self-harm and/or suicidal ideation if they positively endorsed having disclosed to ≥1 person over the past 12 months. We also created a specific disclosure outcome, categorizing respondents as having disclosed to a parent/family member versus a non-family person. Respondents who engaged in non-suicidal self-harm or suicidal ideation were also asked about their online help-seeking behaviour (Supplemental Table S1). Specifically, respondents were asked whether they had looked for help online in the past 12 months to stop harming themselves or to stop thinking about taking their life (yes/no).

#### Sociodemographic Characteristics

We adjusted for several individual- and family-level confounders known to be associated with the exposures and/or outcomes. We controlled for adolescent age (14–15 [referent]/16–17 years) and sex (male [referent]/female). At the family level, we adjusted for low income (no [referent]/yes), educational attainment of the person most knowledgeable about the child (<secondary school [referent], certificate/diploma, and Bachelor's degree or higher), and family structure: two-parent biological/adopted family (no [referent]/yes). Our low-income measure was based on whether the family's before-tax income was above or below the 2013 Statistics Canada low-income cutoff.^25^ A two-parent biological/adopted family structure indicates whether the adolescent resides with married or common-law parents where all children in the household are biological to or adopted by both members of the partner couple.^26^

### Statistical Analyses

Chi-square tests of independence were used for descriptive comparisons. In the main analyses, we used logistic and multinomial logistic regression. Since disclosure and help-seeking were highly prevalent (>10%), we conducted sensitivity analyses using modified Poisson regression modelling to estimate the prevalence ratio (PR). We report both the odds ratio (OR) and PR with their corresponding 95% confidence intervals (95% CI). To obtain provincially representative estimates, we employed survey weights to account for unequal selection probability; weights were adjusted for non-response.^20^ For unbiased variance estimation, we used Statistics Canada-derived bootstrap weights^20^ with a Fay adjustment factor for the mean bootstrap method.^27^ All sample sizes reported are unweighted, while percentages are weighted unless otherwise noted. Missing data across all measures was minimal (*n* = 17) and considered within acceptable limits (<5%_unweighted_). We retained *n* = 342 for analyses, and the analytic sample size varied according to the model due to valid skip patterns (e.g., to be queried regarding online help-seeking for non-suicidal self-harm, a respondent must have positively endorsed self-harm). Logistic and Poisson regression analyses were conducted in SAS (v. 9.4; SAS Institute Inc, Cary, North Carolina) and STATA 15.0 (StataCorp LLC, College Station, TX), respectively.

We conducted a series of sensitivity analyses. The first consisted of adjustment for two additional confounders: child race (Minority or White participant [referent]) and the presence of ≥1 health-professional diagnosed long-term health conditions (e.g., allergies, asthma) (yes/no [referent]). Chronic health conditions were included because they could uniquely impact family dynamics and disclosure and help-seeking behaviours for related mental health concerns, a potential result of healthcare monitoring of the long-term physical health condition. The additional confounders were not included in the primary models because their inclusion, in some cases, would cause us to violate the standard rule of thumb for the minimum ratio of outcome events to predictors (10:1).^28^ While the analytic sample was small by epidemiological standards, requiring us to be mindful of the events:predictors ratio, it is because we restricted study inclusion to participants who positively endorsed self-harm and/or suicidal ideation. Second, to assess the robustness of our findings to missingness, we employed multiple imputation by chained equations^29^ using *m *= 10 imputed datasets, selected based on the proportion of missing and imputation diagnostics: relative increase in variance and the fraction of missing information. All exposures and confounders were included in the imputation model. We did not impute study outcomes; the missingness for these measures was nominal, and exact frequencies could not be released under Statistics Canada data privacy release guidelines. We introduced interaction terms with sex and age (e.g., exposure*sex) for statistically significant (*P *< 0.05) relationships consistent across most models, and assessed model fit using a likelihood ratio test.

### Consent, Standard Approvals, and Data Access

The person most knowledgeable provided informed consent. The University of Ottawa Research Ethics Board (File #H-10-21-7520) approved this study. Statistics Canada approved the study protocol before the investigators were granted data access. Data is publicly available to users with authorized projects through the National Research Data Centres program.^30^

## Results

### Participant Characteristics

Most adolescents were 16–17 years (55.8%) and female (68.5%), while just over half lived in a two-parent biological/adopted family (50.6%) (Supplemental Table S2). More participants reported engaging in non-suicidal self-harm (70.6%) than experiencing suicidal ideation (66.5%), and just over a third reported both (37.0%) (Supplemental Table S3). Disclosure and help-seeking were highly prevalent (Table 1). Of note, disclosure was more prevalent for adolescents who engaged in non-suicidal self-harm (75.6%) than for those who experienced suicidal ideation (67.3%), while seeking help online was more common for adolescents who experienced suicidal ideation (32.8%) than for those who engaged in non-suicidal self-harm (25.6%). While there was variability in the prevalence of disclosure and online help-seeking between females and males (Table 1), there were no statistically significant differences for non-suicidal self-harm or suicidal ideation (*P*s > 0.05).

**Table 1.** Prevalence of Disclosure and Online Help-Seeking Among Adolescents Engaging in Non-Suicidal Self-Harm and/or Experiencing Suicidal Ideation in the 2014 Ontario Child Health Study (*n* = 342).

	Overall		Females		Males	
	Non-suicidal self-harm	Suicidal ideation	Non-suicidal self-harm	Suicidal ideation	Non-suicidal self-harm	Suicidal ideation
	%	%	%	%	%	%
Any disclosure	75.6	67.3	74.6	64.6	78.5	71.8
Specific disclosure						
Told no one	24.4	32.7	25.4	35.4	21.5	28.2
Told non-family	51.3	52.5	51.6	55.2	50.4	47.9
Told parent/family	24.3	14.8	22.9	9.4	28.1	23.9
Sought help online	25.6	32.8	26.8	37.8	22.1	24.4

### Associations Between Family Dynamics and Disclosure and Help-Seeking

Family dysfunction was not associated with overall disclosure of non-suicidal self-harm and suicidal ideation or online help-seeking (Table 2). However, there was some evidence to suggest that family dysfunction may be associated with online help-seeking for suicidal ideation, though the finding was inconsistent (Table 2). In specific disclosure models (Table 3), greater family dysfunction was associated with an increased likelihood of suicidal ideation disclosure to non-family compared to not telling anyone (OR = 1.09, 95% CI: 1.01 to 1.18). In restricted models where disclosure to non-family was the referent, increasing family dysfunction was associated with a reduced likelihood of suicidal ideation disclosure to parents or other family members when compared to non-family (OR = 0.82, 95% CI: 0.71 to 0.94). Poisson and logistic regression models generally agreed, with PR estimates attenuated in magnitude, as expected.

**Table 2.** Relationship Between Family Dysfunction and Disclosure and Online Help-Seeking Among Young Persons Who Engaged in Non-Suicidal Self-Harm and/or Experienced Suicidal Ideation (*n*_analytic_ Range: 198–342).

	Family dysfunction					
Outcome	OR	95% CI	*P*	PR	95% CI	*P*
Disclosure						
Non-suicidal self-harm (NSSH)	1.05	[0.98, 1.13]	0.166	1.01	[1.00, 1.03]	0.161
Suicidal ideation (SI)	1.06	[0.98, 1.15]	0.134	1.02	[0.99, 1.04]	0.136
NSSH and/or SI	1.06	[0.99, 1.12]	0.084	1.01	[1.00, 1.02]	0.060
Online help-seeking						
Non-suicidal self-harm	0.96	[0.91, 1.02]	0.211	0.97	[0.93, 1.02]	0.207
Suicidal ideation	1.08	[1.00, 1.17]	0.059	**1**.**05**	**[1.00, 1.09]**	**0**.**034**
NSSH and/or SI	1.02	[0.96, 1.09]	0.513	1.01	[0.97, 1.06]	0.498

*Note.* Statistically significant findings (*P *< 0.05) appear in **bold**.

**Table 3.** Relationship Between Family Dysfunction and Person-Specific Disclosure of Non-Suicidal Self-Harm and/or Suicidal Ideation (*n*_analytic_ Range: 124–252).

	Family dysfunction					
Specific disclosure	OR	95% CI	*P*	PR	95% CI	*P*
Nominal disclosure models^a^						
Non-suicidal self-harm						
No one	1.00	(REF)		1.00	(REF)	
Non-family	1.06	[0.98, 1.14]	0.126	1.02	[1.00, 1.04]	0.113
Parent/family	1.03	[0.94, 1.14]	0.511	1.02	[0.97, 1.07]	0.353
Suicidal ideation						
No one	1.00	(REF)		1.00	(REF)	
Non-family	**1**.**09**	**[1.01, 1.18]**	**0**.**033**	**1**.**03**	**[1.00, 1.06]**	**0**.**042**
Parent/family	0.93	[0.82, 1.06]	0.263	0.98	[0.90, 1.06]	0.543
Restricted disclosure models^b^						
Non-suicidal self-harm						
Non-family	1.00	(REF)		1.00	(REF)	
Parent/family	0.97	[0.89, 1.06]	0.497	0.98	[0.93, 1.03]	0.452
Suicidal ideation						
Non-family	1.00	(REF)		1.00	(REF)	
Parent/family	**0**.**82**	**[0.71, 0.94]**	**0**.**004**	**0**.**87**	**[0.80, 0.95**]	**0**.**001**

*Note.* Statistically significant findings (*P *< 0.05) appear in **bold**.

^a^ We conducted multinomial logistic regression models where the odds ratio (OR) is reported. Where the prevalence ratio (PR) is reported, we conducted separate modified Poisson regression models when modelling the PR for “non-family” and “parent/family,” using disclosure to “no one” as the referent category (i.e., binary models).

^b^ Restricted to disclosure to non-family (referent) or parent/family.

Positive parenting was not associated with any form of disclosure or online help-seeking for non-suicidal self-harm or suicidal ideation (Tables 4 and 5). One association emerged for negative parenting practices, with greater negative parenting associated with an increased likelihood of online help-seeking for suicidal ideation (OR = 1.22, 95% CI: 1.08 to 1.37). Otherwise, negative parenting practices were not statistically related to disclosure or online help-seeking behaviours (Tables 4 and 5). Findings were consistent between Poisson and logistic regression models.

**Table 4.** Relationship Between Positive and Negative Parenting Practices and Disclosure and Online Help-Seeking Among Young Persons Who Engaged in Non-Suicidal Self-Harm and/or Experienced Suicidal Ideation (*n*_analytic_ Range: 206–349).

	Positive parenting						Negative parenting					
Outcome	OR	95% CI	*P*	PR	95% CI	*P*	OR	95% CI	*P*	PR	95% CI	*P*
Disclosure												
Non-suicidal self-harm (NSSH)	1.03	[0.90, 1.19]	0.655	1.01	[0.97, 1.05]	0.670	1.13	[0.96, 1.33]	0.152	1.03	[0.99, 1.07]	0.166
Suicidal ideation (SI)	1.08	[0.93, 1.24]	0.313	1.02	[0.97, 1.08]	0.342	1.01	[0.90, 1.14]	0.860	1.00	[0.97, 1.04]	0.876
NSSH and/or SI	1.04	[0.93, 1.17]	0.502	1.01	[0.98, 1.04]	0.526	1.03	[0.91, 1.16]	0.665	1.01	[0.98, 1.03]	0.663
Online help-seeking												
Non-suicidal self-harm	1.15	[0.95, 1.39]	0.155	1.11	[0.96, 1.28]	0.153	1.06	[0.92, 1.22]	0.444	1.04	[0.94, 1.15]	0.428
Suicidal ideation	0.94	[0.79, 1.12]	0.501	0.96	[0.87, 1.07]	0.464	**1**.**22**	**[1.08, 1.37]**	**0**.**001**	**1**.**11**	**[1.05, 1.18]**	**<0**.**001**
NSSH and/or SI	0.96	[0.82, 1.13]	0.628	0.97	[0.88, 1.08]	0.623	1.10	[0.99, 1.22]	0.076	1.06	[1.00, 1.12]	0.052

*Note**.*** Statistically significant findings (*P *< 0.05) appear in **bold**.

**Table 5.** Relationship Between Positive and Negative Parenting Practices and Person-Specific Disclosure of non-Suicidal Self-Harm and/or Suicidal Ideation (*n*_analytic_ Range: 130–255).

	Positive parenting						Negative parenting					
Specific disclosure	OR	95% CI	*P*	PR	95% CI	*P*	OR	95% CI	*P*	PR	95% CI	*P*
Nominal disclosure models^a^												
Non-suicidal self-harm												
No one	1.00	(REF)		1.00	(REF)		1.00	(REF)		1.00	(REF)	
Non-family	1.08	[0.92, 1.27]	0.355	1.02	[0.96, 1.09]	0.462	1.09	[0.92, 1.30]	0.334	1.03	[0.97, 1.08]	0.356
Parent/family	0.96	[0.79, 1.15]	0.635	0.99	[0.90, 1.10]	0.897	1.21	[1.00, 1.47]	0.050	1.09	[1.00, 1.18]	0.050
Suicidal ideation												
No one	1.00	(REF)		1.00	(REF)		1.00	(REF)		1.00	(REF)	
Non-family	1.08	[0.92, 1.28]	0.352	1.03	[0.97, 1.10]	0.323	1.01	[0.90, 1.15]	0.823	1.00	[0.97, 1.04]	0.845
Parent/family	1.05	[0.89, 1.25]	0.567	1.04	[0.91, 1.20]	0.557	1.00	[0.87, 1.14]	0.969	0.96	[0.86, 1.08]	0.520
Restricted disclosure models^b^												
Non-suicidal self-harm												
Non-family	1.00	(REF)		1.00	(REF)		1.00	(REF)		1.00	(REF)	
Parent/family	0.88	[0.72, 1.07]	0.212	0.93	[0.84, 1.03]	0.167	1.13	[0.97, 1.32]	0.125	1.07	[0.97, 1.17]	0.189
Suicidal ideation												
Non-family	1.00	(REF)		1.00	(REF)		1.00	(REF)		1.00	(REF)	
Parent/family	0.99	[0.79, 1.23]	0.894	0.99	[0.86, 1.15]	0.932	0.98	[0.84, 1.14][	0.774	0.97	[0.87, 1.09]	0.586

^a^ We conducted multinomial logistic regression models where the odds ratio (OR) is reported. Where the prevalence ratio (PR) is reported, we conducted separate modified Poisson regression models when modelling the PR for “non-family” and “parent/family,” using disclosure to “no one” as the referent category (i.e., binary models).

^b^ Restricted to disclosure to non-family (referent) or parent/family.

### Sensitivity Analyses

Sensitivity analyses (Supplemental Tables 4 to 7) adjusted for child race (Minority: 22.3%, White: 77.7%) and presence of long-term health conditions (Yes: 32.4%, No: 67.6%) mainly yielded similar findings to the main models. The same was true for the multiple imputation results (Supplemental Tables 4 to 7). There was no consistent evidence of interactions with sex and age.

#### Interpretation

Using a provincially representative, population-based survey, we examined relationships between family dysfunction and parenting practices and disclosure and help-seeking behaviours for adolescent non-suicidal self-harm and suicidal ideation. More specifically, we sought to determine whether negative and positive family dynamics experienced at ages 14–17 may influence self-harm and suicidality-related disclosure to parents or other family members and online help-seeking. Overall, our findings suggest that youth living within more negative family environments comprised of family dysfunction and negative parenting practices may be more reluctant to disclose their suicidal thoughts to their parents or other family members and may be more inclined to seek help online for themselves.

While we observed relationships between negative family dynamics and suicidal thoughts, no associations emerged for non-suicidal self-harm. Youth experiencing suicidal ideation may be more likely to disclose their thoughts or seek help online than their self-harm counterparts because of the distressing nature of their thoughts. Psychological distress and suicidal ideation have both been documented to be high among online mental health help-seekers (77% and 75%, respectively).^31^ In contrast, in some cases, youth engaging in non-suicidal self-harm may be less inclined to seek help or disclose their experiences because they perceive the self-harm as a helpful coping strategy, something they do not wish to end. To this end, a systematic review of self-reported reasons for non-suicidal self-harm revealed that self-harm can be viewed as a positive experience, in addition to serving other means, such as managing emotions.^32^ Reasons for self-harm to be viewed positively include the behaviours being perceived as gratifying or comforting to the self-injurer.^32^ Motivations for self-harm and suicidality are complex, and differences in the functions they serve may have affected our findings.

Contrary to previous work, which suggests lower rates of disclosure among adolescents,^13,33^ we observed high rates of disclosure for both non-suicidal self-harm and suicidal ideation. However, disclosure to non-family was more common than telling one's parent(s) or another family member, a finding substantiated in our regression modelling. As family dysfunction increased, youth experiencing suicidal ideation were more likely to disclose their suicidal thoughts to non-family than not tell anyone and were less likely to disclose to parents/family than non-family. This pattern of findings aligns with other work demonstrating that peer-based social support increases with age among adolescents experiencing suicidal ideation,^34^ though we should note that disclosure to peers was one category of other persons included in our operationalization of non-family. Regardless, disclosure to non-family may represent an important first step on a recovery journey (acknowledgement and sharing). The high prevalence of disclosure within our sample could be explained by the beneficial influence of population-level mental health anti-stigma campaigns (e.g., a Canada-wide campaign that commenced in September 2010)^35^ that have also been speculated to have led to increased care seeking, ultimately increasing utilization rates of acute services.^8^ Consistent with earlier studies, we observed that help-seeking was less common than disclosure, suggesting that the behaviours may functionally differ.^33^ Help-seeking is often conceptualized as a purposeful behaviour intended to solicit support, whereas disclosure may occur without help-seeking intent.^33^ Online help-seeking, as captured by the present study, may represent a more formal willingness or readiness on behalf of the youth to want to get well, especially in the context of negative family dynamics where perhaps they feel they have less support and may need to be self-directed. In line with this, we observed robust relationships between negative parenting practices and online help-seeking for suicidal ideation and an inconsistent association for family dysfunction.

The family environment is a commonly identified way to help young persons who self-harm.^36^ Specific suggestions on ways parents can help children after they have engaged in non-suicidal self-harm include offering more emotional support, reducing conflict, and engaging in family-orientated leisure-time activities.^36^ These identified themes of how the family environment can be leveraged to support a child post-self-harm are the same features of positive and negative parenting practices and family functioning included in this study. These are also aspects of the family environment that have been longitudinally implicated in the onset of adolescent suicidality.^37^ Kingsbury et al.^37^ identified prospective, direct relationships between harsh but not positive parenting practices and the onset of suicidal ideation and attempt at ages 14–15. Thus, addressing family dynamics could also have more upstream impact, expanding their utility beyond post-self-harm support. Our demonstrated associations between higher levels of family dysfunction and a reduced likelihood of disclosure to parents or other family members for suicidal ideation suggests that reducing conflict and discord within the family, thereby increasing family functioning, may lead to increased sharing of suicidal thoughts, supporting youth suggestions to minimize conflict and improve communication as a means to help post-self-harm.^36^ Negative family dynamics may represent an under-considered barrier to disclosing self-harm and suicidal thoughts to individuals who may be in the best situation to facilitate professional help-seeking: parents and family.

Finally, we did not observe any relationships suggesting a link between positive parenting strategies and disclosure or online help-seeking. The unobserved associations agree with another study that found that positive parental authoritativeness and social support did not longitudinally predict future help-seeking behaviour for general mental health concerns.^17^ We should note that both positive and negative parenting practices were not related to the disclosure of self-harm or suicidal thoughts. Disclosure behaviours can serve multiple purposes, such as seeking peer validation,^33^ thus, the absence of statistical associations in the present study could be explained by heterogeneity in the outcome. Unfortunately, there is limited literature on the function or intention behind self-harm disclosure,^33^ and the OCHS did not capture information on the motivation behind this behaviour. Considering the observed associations between family dysfunction and disclosure, we may also conclude that family functioning may have a greater impact on a child's willingness to share information. Unlike parenting practices, family functioning captures the everyday functioning of the entire family unit, including aspects of communication and acceptance, such as feeling accepted for who they are, being able to discuss fears or concerns, and expressing feelings.^16^ Family dysfunction may represent a chronic daily stressor for adolescents, which could deter them from sharing personal information with their parent/caregiver, especially when it pertains to sensitive mental health concerns. Negative aspects of the family environment may be most influential in affecting disclosure and help-seeking behaviours for serious mental health concerns of adolescent suicidality.

### Limitations and Future Advancements

While the overall survey response rate was low (50.8%), it reflects current population-based survey challenges.^30^ To highlight survey administration challenges, a recent comparable national survey of similar-aged peers had a much lower (41.3%) response rate.^38^ The low response rate may have affected statistical power. This concern may be exacerbated here since study inclusion was further restricted to participants who were surveyed about sensitive items such as self-harm (14–17 years) and who positively endorsed self-harm and/or suicidal ideation. Our potential selection bias concerns were mitigated by using the OCHS sampling weights, which were adjusted for non-response.^30^

Another limitation is that our disclosure outcome did not differentiate between self-disclosure and discovery, a key distinction noted in the literature.^33^ In the present study, youth reported whether they had told anyone. They were not explicitly asked to indicate whether this was a voluntary sharing of information or whether they were forced to acknowledge their behaviours because someone else noticed something (e.g., self-inflicted injury) or their self-harm required medical attention. However, the discovery of the self-harm is likely somewhat mitigated in this context because participants were asked whether they told anyone rather than being asked if anyone *knew* about their self-harm (with or without the adolescent voluntarily communicating). Although, parental awareness of child self-harm may have affected the measurement of our exposures. When a parent is aware of their child's self-harm, ratings of family dysfunction are higher.^39^ Though when compared to adolescent reports, parental ratings of family dysfunction are lower regardless of whether the parent is aware of the self-harm.^39^ Thus, compared to the potential for adolescent self-report, we expect parental awareness and recall bias to have minimal impact on exposure ascertainment. It is also possible that parental awareness of self-harm may destabilize the family environment, affecting family discord and conflict.^40^ Undoubtedly, an advancement of future work would be to assess relationships prospectively, capturing measurement of family dynamics before the onset of self-harm or suicidal thoughts. Finally, because the general functioning subscale of the McMaster Family Assessment Device^16^ was used, we could not explore which aspects of family dysfunction (e.g., impairments in affective responsiveness) were most relevant.

## Conclusion

Adolescents exposed to negative family dynamics, specifically family dysfunction and negative parenting practices, may be less inclined to disclose their suicidal thoughts to parents and family and instead be more likely to look for help online. While help-seeking online may yield beneficial results, navigating the internet and obtaining professional support as an adolescent may be challenging. Well-maintained, evidence-based online resources for support-seeking youth are urgently needed; however, parents and family may be some of the best facilitators of professional help-seeking and integral to adolescent recovery. A reduced willingness to disclose severe mental health concerns to these individuals could potentially prevent their support until acute care is required. Identifying family-level factors that may encourage help-seeking could lead to their incorporation as part of mental health promotion strategies to mitigate self-harm recurrence or prevent suicide attempts. At the very least, mental health literacy campaigns should have active components on what to do when someone discloses or seeks support for their self-harm or suicidal ideation. The role of negative family dynamics as a barrier to mental health support-seeking may be overlooked.

## Supplemental Material

sj-docx-1-cpa-10.1177_07067437251315526 - Supplemental material for The Relationship Between Family Dynamics and Help-Seeking and Disclosure of Adolescent Self-Harm and Suicidality: A Population-Representative Study: Relation entre dynamique familiale et recherche d’aide, et dévoilement des actes d’automutilation et de la suicidalité chez les adolescents : étude représentative de la population Supplemental material, sj-docx-1-cpa-10.1177_07067437251315526 for The Relationship Between Family Dynamics and Help-Seeking and Disclosure of Adolescent Self-Harm and Suicidality: A Population-Representative Study: Relation entre dynamique familiale et recherche d’aide, et dévoilement des actes d’automutilation et de la suicidalité chez les adolescents : étude représentative de la population by Nicole G. Hammond, Christopher Gravel, Mark A. Ferro, Hannah Landry, Marie-Claude Geoffroy, Nicole Racine and Ian Colman in The Canadian Journal of Psychiatry
