# Supplementary material for: The Relationship Between Family Dynamics and Help-Seeking and Disclosure of Adolescent Self-Harm and Suicidality: A Population-Representative Study: Relation entre dynamique familiale et recherche d’aide, et dévoilement des actes d’automutilation et de la suicidalité chez les adolescents : étude représentative de la population
Source: Can J Psychiatry. 2025 Feb 3;70(5):404–13. doi: 10.1177/07067437251315526 (PMC11795579; doi:10.1177/07067437251315526)
Supplement: sj-docx-1-cpa-10.1177_07067437251315526 - Supplemental material for The Relationship Between Family Dynamics and Help-Seeking and Disclosure of Adolescent Self-Harm and Suicidality: A Population-Representative Study: Relation entre dynamique familiale et recherche d’aide, et dévoilement des actes d’ [file sj-docx-1-cpa-10.1177_07067437251315526.docx]

| **Supplemental eTable 1.** Study-specific mental health questions from the 2014 Ontario Child Health Study (2014 OCHS). | |
| --- | --- |
| **Measure name** | **Survey questions**^1^ |
| **Non-suicidal self-harm** | “Sometimes people deliberately harm themselves but they do not mean to take their life. In the **past 12 months**, did you ever deliberately harm yourself but not mean to take your life?”   - “Yes” - “No” |
| **Suicidal ideation** | “In the **past 12 months**, did you ever **seriously** consider taking your own life or killing yourself?”   - “Yes” - “No” |
| **Disclosure of non-suicidal self-harm** | “In the **past 12 months**, did you **tell any of the following people** that you harmed yourself?”   1. “parent or other family member” (yes/no) 2. “friend or partner” (yes/no) 3. “teacher or other adult at school” (yes/no) 4. “doctor, nurse or mental health professional (e.g., psychologist, social worker, counsellor)” (yes/no) 5. “someone on a phone helpline or crisis hotline” (yes/no) 6. “someone on the internet” (yes/no) 7. “a minister, priest, rabbi, Imam, or other spiritual leader” (yes/no) 8. “naturopath, herbalist or alternative practitioner” (yes/no) 9. “I told someone else” (yes/no) |
| **Disclosure of suicidal ideation** | “In the **past 12 months**, did you ever **tell any of the following people** that you were seriously considering taking your own life or killing yourself?”   1. “parent or other family member” (yes/no) 2. “friend or partner” (yes/no) 3. “teacher or other adult at school” (yes/no) 4. “doctor, nurse or mental health professional (e.g., psychologist, social worker, counsellor)” (yes/no) 5. “someone on a phone helpline or crisis hotline” (yes/no) 6. “someone on the internet” (yes/no) 7. “a minister, priest, rabbi, Imam, or other spiritual leader” (yes/no) 8. “naturopath, herbalist or alternative practitioner” (yes/no) 9. “I told someone else” (yes/no) |
| **Online help-seeking for non-suicidal self-harm** | “In the **past 12 months**, did you ever look for help on the internet to stop harming yourself?”   - “Yes” - “No” |
| **Online help-seeking for suicidal ideation** | “In the **past 12 months**, did you ever look for help on the internet for ways to stop thinking about taking your  own life?”   - “Yes” - “No” |
| **Reference**  1. Ontario Child Health Study. 2014 Ontario Child Health Study Adolescent Questionnaire (Ages 14-17). Published online July 2014. Accessed December 6, 2023. https://ontariochildhealthstudy.s3.amazonaws.com/uploads/2014OCHS_Adolescent_14-17YRS_July2014.pdf | |

| **Supplemental eTable 2.** Characteristics of adolescents 14-17 years in the 2014 Ontario Child Health Study who reported non-suicidal self-harm and/or suicidal ideation (n=342). | |
| --- | --- |
| **Characteristic** | **%** |
| Age |  |
| 14-15 years | 44.2 |
| 16-17 years | 55.8 |
| Sex |  |
| Male | 31.5 |
| Female | 68.5 |
| Low income |  |
| No | 82.7 |
| Yes | 17.3 |
| Parental education |  |
| <Secondary school | 21.2 |
| Certificate/diploma | 55.9 |
| ≥Bachelor degree | 22.8 |
| Intact family |  |
| No | 49.4 |
| Yes | 50.6 |

| **Supplemental eTable 3.** Self-harm and suicidal ideation characteristics of adolescents engaging in non-suicidal self-harm and/or experiencing suicidal ideation in the 2014 Ontario Child Health Study (n=342). | |
| --- | --- |
|  | % |
| **Non-suicidal self-harm (nssh)** | 70.6 |
| Frequency of nssh |  |
| One time | 42.5 |
| More than once | 57.5 |
| Most common forms of nssh |  |
| Self-cutting or scratching | 84.6 |
| Other | 12.4 |
| **Suicidal ideation** | 66.5 |
| Suicide plan | 48.0 |
| Suicide attempts |  |
| One time | 29.1 |
| More than once | 25.1 |
| **Combined nssh and suicidal ideation** | 37.0 |

| **Supplemental eTable 4.** Relationship between family dysfunction and disclosure and online help-seeking among young persons who engaged in non-suicidal self-harm and/or experienced suicidal ideation. | | | | | | |
| --- | --- | --- | --- | --- | --- | --- |
|  | **Family dysfunction** | | | | | |
|  | Additional confounders^a^ | | | Multiple imputation | | |
| Outcome | OR | 95% CI | *P* | PR | 95% CI | *P* |
| **Disclosure** |  |  |  |  |  |  |
| Non-suicidal self-harm (NSSH) | 1.05 | 0.97, 1.14 | 0.215 | 1.01 | 1.00, 1.03 | 0.155 |
| Suicidal ideation (SI) | 1.06 | 0.97, 1.15 | 0.212 | 1.02 | 0.99, 1.04 | 0.158 |
| NSSH and/or SI | 1.05 | 0.99, 1.12 | 0.109 | 1.01 | 1.00, 1.03 | 0.094 |
| **Online help-seeking** |  |  |  |  |  |  |
| Non-suicidal self-harm | 0.94 | 0.88, 1.01 | 0.093 | 0.97 | 0.93, 1.02 | 0.296 |
| Suicidal ideation | 1.08 | 1.00, 1.18 | 0.060 | **1.05** | **1.00, 1.09** | **0.049** |
| NSSH and/or SI | 1.02 | 0.95, 1.09 | 0.563 | 1.01 | 0.97, 1.05 | 0.571 |
| ***Note.*** Statistically significant findings (*P*<0.05) appear in bold.  ^a^Additionally adjusted for child race and the presence of long-term health conditions (n_analytic_ range: 188-318). | | | | | | |

| **Supplemental eTable 5.** Relationship between family dysfunction and person-specific disclosure of non-suicidal self-harm and suicidal ideation. | | | | | | |
| --- | --- | --- | --- | --- | --- | --- |
|  | **Family dysfunction** | | | | | |
|  | Additional confounders^a^ | | | Multiple imputation | | |
| Specific disclosure | OR | 95% CI | *P* | PR | 95% CI | *P* |
| ***Nominal disclosure models^b^*** |  |  |  |  |  |  |
| Non-suicidal self-harm |  |  |  |  |  |  |
| No one | 1.00 | (REF) |  | 1.00 | (REF) |  |
| Non-family | 1.07 | 0.99, 1.15 | 0.094 | 1.02 | 1.00, 1.04 | 0.120 |
| Parent/ family | 1.02 | 0.91, 1.15 | 0.746 | 1.02 | 0.97, 1.08 | 0.338 |
| Suicidal ideation |  |  |  |  |  |  |
| No one | 1.00 | (REF) |  | 1.00 | (REF) |  |
| Non-family | 1.08 | 0.99, 1.18 | 0.070 | 1.03 | 1.00, 1.06 | 0.062 |
| Parent/ family | 0.92 | 0.80, 1.05 | 0.226 | 0.98 | 0.91, 1.05 | 0.530 |
| ***Restricted disclosure models^c^*** |  |  |  |  |  |  |
| Non-suicidal self-harm |  |  |  |  |  |  |
| Non-family | 1.00 | (REF) |  | 1.00 | (REF) |  |
| Parent/ family | 0.96 | 0.86, 1.06 | 0.388 | 0.98 | 0.93, 1.03 | 0.481 |
| Suicidal ideation |  |  |  |  |  |  |
| Non-family | 1.00 | (REF) |  | 1.00 | (REF) |  |
| Parent/ family | **0.82** | **0.72, 0.95** | **0.006** | **0.88** | **0.81, 0.95** | **0.002** |
| ***Note.*** Statistically significant findings (*P*<0.05) appear in bold.  ^a^We conducted multinomial logistic regression models where the odds ratio (OR) is reported. Where the prevalence ratio (PR) is reported, we conducted separate modified Poisson regression models when modelling the PR for “non-family” and “parent/family”, using disclosure to “no one” as the referent category (i.e., binary models).  ^b^Restricted to disclosure to non-family (referent) or parent/ family.  ^c^Additionally adjusted for child race and presence of long-term health conditions (n_analytic_ range: 118-233). | | | | | | |

| **Supplemental eTable 6.** Relationship between positive and negative parenting practices and disclosure and online help-seeking among young persons who engaged in non-suicidal self-harm and/or experienced suicidal ideation. | | | | | | | | | | | | |
| --- | --- | --- | --- | --- | --- | --- | --- | --- | --- | --- | --- | --- |
|  | **Positive parenting** | | | | | | **Negative parenting** | | | | | |
|  | Additional confounders^a^ | | | Multiple imputation | | | Additional confounders^a^ | | | Multiple imputation | | |
| Outcome | OR | 95% CI | *P* | PR | 95% CI | *P* | OR | 95% CI | *P* | PR | 95% CI | *P* |
| **Disclosure** |  |  |  |  |  |  |  |  |  |  |  |  |
| Non-suicidal self-harm (NSSH) | 1.01 | 0.89, 1.16 | 0.854 | 1.01 | 0.97, 1.05 | 0.773 | 1.08 | 0.91, 1.29 | 0.389 | 1.04 | 1.00, 1.08 | 0.069 |
| Suicidal ideation (SI) | 1.08 | 0.94, 1.25 | 0.271 | 1.02 | 0.97, 1.07 | 0.438 | 0.97 | 0.86, 1.09 | 0.607 | 1.01 | 0.98, 1.04 | 0.597 |
| NSSH and/or SI | 1.03 | 0.93, 1.16 | 0.561 | 1.01 | 0.98, 1.04 | 0.645 | 1.01 | 0.89, 1.15 | 0.867 | 1.01 | 0.99, 1.04 | 0.375 |
| **Online help-seeking** |  |  |  |  |  |  |  |  |  |  |  |  |
| Non-suicidal self-harm | 1.15 | 0.95, 1.39 | 0.163 | 1.10 | 0.95, 1.26 | 0.192 | 1.06 | 0.92, 1.21 | 0.447 | 1.05 | 0.94, 1.17 | 0.380 |
| Suicidal ideation | 0.94 | 0.81, 1.09 | 0.417 | 0.96 | 0.86, 1.06 | 0.433 | **1.22** | **1.07, 1.39** | **0.002** | **1.12** | **1.06, 1.19** | **<0.001** |
| NSSH and/or SI | 0.94 | 0.81, 1.09 | 0.430 | 0.98 | 0.88, 1.08 | 0.663 | 1.10 | 0.99, 1.22 | 0.076 | **1.07** | **1.00, 1.13** | **0.049** |
| ***Note.*** Statistically significant findings (*P*<0.05) appear in bold.  ^a^Additionally adjusted for child race and presence of long-term health conditions (n_analytic_ range: 195-325). | | | | | | | | | | | | |

| **Supplemental eTable 7.** Relationship between positive and negative parenting practices and person-specific disclosure of non-suicidal self-harm and suicidal ideation. | | | | | | | | | | | | |
| --- | --- | --- | --- | --- | --- | --- | --- | --- | --- | --- | --- | --- |
|  | **Positive parenting** | | | | | | **Negative parenting** | | | | | |
|  | Additional confounders^a^ | | | Multiple imputation | | | Additional confounders^a^ | | | Multiple imputation | | |
| Specific disclosure | OR | 95% CI | *P* | PR | 95% CI | *P* | OR | 95% CI | *P* | PR | 95% CI | *P* |
| ***Nominal disclosure models^b^*** |  |  |  |  |  |  |  |  |  |  |  |  |
| Non-suicidal self-harm |  |  |  |  |  |  |  |  |  |  |  |  |
| No one | 1.00 | (REF) |  | 1.00 | (REF) |  | 1.00 | (REF) |  | 1.00 | (REF) |  |
| Non-family | 1.05 | 0.89, 1.24 | 0.540 | 1.02 | 0.96, 1.08 | 0.539 | 1.06 | 0.88, 1.28 | 0.552 | 1.03 | 0.98, 1.09 | 0.265 |
| Parent/ family | 0.91 | 0.75, 1.10 | 0.326 | 0.98 | 0.89, 1.08 | 0.711 | 1.16 | 0.93, 1.43 | 0.185 | **1.10** | **1.02, 1.20** | **0.018** |
| Suicidal ideation |  |  |  |  |  |  |  |  |  |  |  |  |
| No one | 1.00 | (REF) |  | 1.00 | (REF) |  | 1.00 | (REF) |  | 1.00 | (REF) |  |
| Non-family | 1.09 | 0.92, 1.28 | 0.318 | 1.03 | 0.97, 1.09 | 0.407 | 0.97 | 0.85, 1.10 | 0.643 | 1.01 | 0.97, 1.05 | 0.580 |
| Parent/ family | 1.06 | 0.87, 1.30 | 0.564 | 1.02 | 0.90, 1.16 | 0.723 | 0.98 | 0.84, 1.13 | 0.750 | 0.97 | 0.87, 1.09 | 0.652 |
| ***Restricted disclosure models^c^*** |  |  |  |  |  |  |  |  |  |  |  |  |
| Non-suicidal self-harm |  |  |  |  |  |  |  |  |  |  |  |  |
| Non-family | 1.00 | (REF) |  | 1.00 | (REF) |  | 1.00 | (REF) |  | 1.00 | (REF) |  |
| Parent/ family | 0.85 | 0.67, 1.08 | 0.185 | 0.93 | 0.85, 1.03 | 0.170 | 1.10 | 0.94, 1.28 | 0.243 | 1.07 | 0.97, 1.18 | 0.184 |
| Suicidal ideation |  |  |  |  |  |  |  |  |  |  |  |  |
| Non-family | 1.00 | (REF) |  | 1.00 | (REF) |  | 1.00 | (REF) |  | 1.00 | (REF) |  |
| Parent/ family | 0.99 | 0.76, 1.30 | 0.966 | 0.99 | 0.86, 1.14 | 0.885 | 1.01 | 0.86, 1.18 | 0.951 | 0.97 | 0.86, 1.09 | 0.613 |
| ***Note.*** Statistically significant findings (*P*<0.05) appear in bold.  ^a^We conducted multinomial logistic regression models where the odds ratio (OR) is reported. Where the prevalence ratio (PR) is reported, we conducted separate modified Poisson regression models when modelling the PR for “non-family” and “parent/family”, using disclosure to “no one” as the referent category (i.e., binary models).  ^b^Restricted to disclosure to non-family (referent) or parent/ family.  ^c^Additionally adjusted for child race and the presence of long-term health conditions (n_analytic_ range: 123-236). | | | | | | | | | | | | |
